# Increased tumor-infiltrating plasmacytoid dendritic cells promote cancer cell proliferation and invasion via TNF-α/NF-κB/CXCR-4 pathway in oral squamous cell carcinoma

**DOI:** 10.7150/jca.55580

**Published:** 2021-03-19

**Authors:** Nannan Han, Xing Li, Yupu Wang, Lin Wang, Chunye Zhang, Zun Zhang, Min Ruan, Chenping Zhang

**Affiliations:** 1 Department of Oral Maxillofacial-Head and Neck Oncology, Shanghai Ninth People's Hospital, College of Stomatology, Shanghai Jiao Tong University School of Medicine, Shanghai, 200011, China.; 2 Shanghai Key Laboratory of Stomatology, Shanghai Research Institute of Stomatology, National Clinical Research Center for Oral Diseases, Shanghai, 200011, China.; 3 Department of Dentistry, Affiliated Hospital, Weifang Medical University, Weifang, 261031, China.; 4 Department of Oral Pathology, Shanghai Ninth People's Hospital, College of Stomatology, Shanghai Jiao Tong University School of Medicine, Shanghai, 200011, China.; 5 Department of Stomatology, Shanghai East Hospital, Tongji University. Shanghai 200120, China.

**Keywords:** oral squamous cell carcinoma (OSCC), plasmacytoid dendritic cells (pDCs), lymph node metastasis, nuclear factor-κB (NF-κB), chemotaxis cytokine receptor-4 (CXCR-4)

## Abstract

**Background:** Tumor-infiltrating immune cells are closely associated with tumor occurrence and progression. The present study explored the potential mechanism of tumor-infiltrating plasmacytoid dendritic cells (pDC) mediating the proliferation and metastasis of cancer cells in oral squamous cell carcinoma (OSCC).

**Methods:** pDC distribution was detected by immunofluorescence and flow cytometry. chemotaxis cytokine receptor-4/7 (CXCR-4/7) expression was detected by quantitative polymerase chain reaction and immunohistochemistry. Cell proliferation and migration were measured by CCK-8, colony formation, wound healing and transwell assay. ELISA and western blotting were used to investigate cytokines secretion and NF-κB pathway activity.

**Results:** Tumor-infiltrating pDC in OSCC was significantly increased and associated with tumor size, lymph node (LN) metastasis (*P* <0.05). Tumor-infiltrating-pDC-conditioned medium from OSCC patients significantly promoted tumor cell proliferation and invasion, which was at least partly mediated via enhancing the CXCR-4 expression of tumor cell. In addition, the activation of NF-κB pathway played a decisive role in the overexpression of CXCR-4, which was further regulated by pDC-derived TNF-α secretion.

**Conclusions:** Tumor-infiltrating pDC promoted oral cancer proliferation and invasion via activating the TNF-α/NF-κB/CXCR-4 pathway, which may serve as a potential immunological target for the treatment of OSCC in the future.

## Introduction

Oral squamous cell carcinoma (OSCC) is the most frequent histological type of head and neck cancer and the sixth most frequent malignancy across the world, with over 800,000 newly diagnosed cases and 166,000 people dying each year 1, 2. Despite new therapeutic modalities being introduced over the past two decades, the overall 5-year survival rate for OSCC remains only approximately 60%, highlighting the urgent need to identify new approaches for the prevention and treatment of OSCC 2, 3. In recent years, much attention has been directed toward tumor immunology research, with a particular focus on tumor-infiltrating immune cells 4, 5.

Tumor-infiltrating immune cells gather in the tumor microenvironment, accompanying cancer occurrence and progression 6. Growing evidence suggests that the complex function of these cells is important in explaining the development, growth, and metastasis of cancer, besides the features of cancer cells themselves 7, 8. However, in the past decades, the major research focus regarding tumor-infiltrating immune cells has been the tumor-infiltrating lymphocytes, including CD4^+^ T cells, CD8^+^ T cells, and regulatory T cells, while the role of antigen-presenting cells, such as myeloid dendritic cells (mDC) and plasmacytoid dendritic cells (pDC), has not been widely explored 9-11.

mDC and pDC are two of the main professional antigen-presenting cells and play a critical, decisive role in determining the outcome of the immune response to antigens 12. Recent studies suggest that pDC, specifically, are rather important in cancer immunity as these cells have been identified in many solid malignant tumors, including those of head and neck, breast, ovarian, and lung cancers 13, 14. Unfortunately, pDC recruited to the tumor microenvironment often displays a non-activated state and is associated with tumor progression 15. Further, in breast tumors, infiltration of pDC in the primary lesion was found to be correlated with adverse outcomes 16, 17; while in ovarian cancer, the presence of pDC was not only associated with a poor clinical outcome but also served as a predictor of early relapse 18.

Previously, we also demonstrated an increased frequency of tumor-infiltrating pDC in the primary OSCC microenvironment 19. Further molecular investigation showed that oral cancer cells could down-regulate TLR-9-mediated interferon-α (IFN-α) production in pDC via increasing TGF-β and IL-10 secretion, which may lead to severe functional impairment of these infiltrated pDC 20. In addition, our clinicopathologic analysis revealed that an increased number of tumor-infiltrating pDC has a tendency to correlate with LN metastasis (*p* < 0.05), suggesting that these impaired pDC may facilitate cancer cells migration and invasion 19; however, the underlying molecular mechanism remains unknown. The aim of this study sought to clarify the potential mechanism by which pDC mediated cancer cell proliferation and lymph node metastasis in oral squamous cell carcinoma.

## Methods

### Patients and samples

A total of 122 patients with primary OSCC treated between January 2014 and November 2014 in the Department of Oral Maxillofacial-Head and Neck Oncology, Ninth People's Hospital, Shanghai Jiao Tong University School of Medicine, were enrolled. Patients fulfilling the following criteria were included in the study: (1) primary OSCC confirmed by pathology after surgery; (2) no radiotherapy, chemotherapy, or immunotherapy before surgery; and (3) complete clinicopathological data available. Paraffin-embedded tumor tissue sections prepared for immunohistochemistry of CD123.

Subsequently, from January 2018 to July 2018, we collected an additional 60 tumor tissue samples from OSCC patients that met the above criteria. Six patients (three males and three females, mean age of 57.2 years old) who underwent alveoloplasty were selected as controls. The collected tumor samples and normal oral mucosal epithelium were quickly washed in phosphate-buffered saline (PBS) thrice and divided into three portions, one of which was soaked in formalin, another was soaked in high-glucose Dulbecco's Modified Eagle Medium (DMEM; Gibco, Invitrogen, Carlsbad, USA) supplemented with 10% fetal bovine serum (FBS; Biowest, France) and the last was immediately frozen in liquid nitrogen and stored at -80 °C for further analysis. No significant differences were noted in age, gender, alcohol use, or smoking habits between the control and OSCC groups. This study was approved by the Shanghai Ninth People's Hospital IRB and informed signed consent was obtained from each patient.

### Single-cell suspension preparation

Surgical human primary OSCC tissue samples were washed several times with PBS and carefully minced to small pieces in sterile serum-free high-glucose DMEM (supplemented with 100 U/mL penicillin, 1 mM glutamine, and 100 U/mL streptomycin). Tumor tissue was digested with collagenase type VIII (1.5 mg/mL; Sigma, USA) and DNase type I (1 mg/mL; Sigma, USA) for 120 min at 37 °C with gentle agitation. The resulting cell suspensions were washed in PBS, resuspended in PBS containing trypsin/EDTA, and filtered through a 40-mm nylon cell strainer (Falcon, Becton Dickinson Labware) into cold DMEM containing 10% FBS.

### Isolation of pDC and preparation of pDC-conditioned medium (CM)

pDC were isolated using magnetically activated cell sorting with the BDCA-4 dendritic cell isolation kit from Miltenyi Biotec according to manufacturer's instructions^21^. pDC was labeled with anti-BDCA-4 antibody coupled to colloidal paramagnetic microbeads and passed through magnetic separation columns twice (LS and RS columns; Miltenyi Biotec). The purity of the isolated pDC (APC-conjugated anti-CD123^+^ and PERCP-conjugated anti-MHC II^+^) was >90%. Viability was >95% as determined using the trypan blue exclusion test. Isolated pDC were seeded at a final concentration of 5 × 10^5^ cells/mL and cultured overnight in serum-free DMEM at 37 °C and 5% CO_2_ to prepare pDC‑conditioned medium (pDC-CM). pDC were then collected by centrifugation at 3000 rpm for further experiments, and pDC-CM were collected and stored at -80 °C for* in vitro* cancer cell line stimulation.

### Cell culture and stimulation

For cell line stimulation, CAL 27 were purchased from American Type Culture Collection (ATCC^®^ CRL-2095) and the human HNSCC cell lines, HN 30, were obtained from the National Institutes of Health (Rockville, MD, USA). HN 30 cells were seeded at a density of 4 × 10^5^ and cultured in DMEM supplemented with 10% FBS in a humidified atmosphere of 5% CO_2_, at 37 °C for 24 h. The cells were washed twice with 1× PBS and starved in 1% FBS-supplemented growth medium overnight. Next, cells were stimulated with different concentrations of pDC-CM, with or without anti-TNF-α antibodies or TNF-α (50 ng/mL; R&D Systems, USA) for 72 h. Experiments were repeated at a minimum of three times.

Lentiviraus-CXCR-4-shRNA was constructed at Gene Pharma (Shanghai, China). Cells were plated in 6‐well plates and infected with lentivirus for 24 h in a complete medium containing 5 μg/ml polybrene.

### Immunohistochemical staining (IHC)

Formalin-fixed, paraffin-embedded tissue blocks from OSCC patients were used. IHC was performed on 4-mm-thick routinely processed paraffin sections, according to a standard protocol. After deparaffinizing and rehydrating, tissue sections were incubated in sodium citrate buffer and heated for antigen retrieval. anti-CXCR-4 antibody (1:150; Rabbit; Proteintech, USA), or anti-CXCR-7 antibody (1:200; Rabbit; Proteintech, USA) was applied to tissue sections overnight at 4 °C. All sections were then incubated with a corresponding secondary antibody for 30 min at 23-25 °C. Immunohistochemical evaluations were performed independently by two pathologists who were not informed of the clinicopathological profile of the patients. CXCR-4/7 expressions were quantified using a visual grading system as previously reported 22. According to staining intensity and percentage of positive tumor cells, the final staining score was given: 0 (negative); 1 (<50% weak or strong positive cells); 2 (>50% weak positive cells); 3 (>50% strong positive cells). For statistical analysis, score (0, 1) and (2, 3) were considered as low and high expression, respectively.

### Immunofluorescent staining

Tissue sections were deparaffinized and rehydrated with xylene, gradient ethanol, and distilled water. After rinsing thrice with PBS, the tissue sections were blocked with 3% BSA for 30 min and incubated with anti-CD123 antibody (10 µg/mL; goat; R&D Systems, USA) overnight at 4 °C. Tissue sections were then washed and incubated for 30 min with an Alexa Fluor 488-conjugated anti-mouse IgG F(ab') 2 fragment (1:200; Invitrogen, USA). Cells were co-stained with 4′,6-diamidino-2-phenylindole (1:300; Invitrogen) to detect nuclei, after which they were observed and imaged using a TCS SP2 laser-scanning confocal microscope (Leica Microsystems, Germany). Based on CD123 staining, pDC infiltrates were considered low if < 10 pDC per high-power field (HPF, 400×) were observed, high if > 10 pDC per HPF were observed 23.

### Flow cytometry

Flow cytometric analysis was performed as previously described by Hartmann et al. 21. Briefly, cells were incubated at 4 °C for 30 min in PBS with 0.1% BSA and 0.01% NaN_3_ in the presence of appropriately diluted labeled monoclonal antibodies, which were purchased from BD Pharmingen (USA) and used as follows: APC-conjugated anti-CD123^+^ and PERCP-conjugated anti-MHC II^+^. The cells were subsequently analyzed using a FACSCalibur and CellQuest FACS analysis software (BD Biosciences, Erembodegem-Aalst, Belgium).

### Cytokine production analysis

The Bio-Plex Cytokine Assay (Bio-Rad, Munich, Germany) was used for detection of IL-6, IL-8, and TNF-α. An IFN-α module set from Bender Med Systems (Vienna, Austria) was used to detect IFN-α in cell culture supernatants, according to the manufacturer's instructions. These cytokine assays allowed for the multiplexed quantitative measurement of multiple cytokines in a small volume of cell culture supernatant. The protein array was analyzed by a dedicated microplate reader system (Bio-Plex Array Reader; Bio-Rad) and data were calculated by the Bio-Plex Manager software. Experiments were repeated at a minimum of three times.

### Quantitative real‑time PCR (qPCR)

The preparation of total RNA and cDNA was performed as previously described 24. CXCR-4 (primer sequences: upstream, 5'-ACTACACCGAGGAAATGGGCT-3'; downstream, 5'-GCTACCCACAATGCCAGTTAAGAAGA-3'), CXCR-7 (primer sequences: upstream, 5'-TCTGCATCTCTTCGACTACTCA-3'; downstream, 5'-GTAGAGCAGGACGCTTTTGTT-3'), and glyceraldehyde 3-phosphate dehydrogenase (GAPDH; primer sequences: upstream, 5'- GGAGCGAGATCCCTCCAAAAT-3', downstream; 5'- GGCTGTTGTCATACTTCTCATGG-3') mRNA were detected by real-time PCR using the TaqMan^TM^ Gene Expression Assay (Applied Biosystems, Life Technologies Life Technologies, USA). Gene-specific products were measured continuously by an ABI PRISM 7000 Sequence Detection System (Applied Biosystems) over 40 cycles. Experiments were repeated at a minimum of three times.

### Western blotting (WB)

Cells were treated with lysis buffer (PBS containing 1% Triton X-100, protease inhibitor cocktail, and 1 mmol/L phenylmethylsulfonyl fluoride) at 4 °C for 30 min. Equal concentrations of protein were subjected to SDS-PAGE. Following transfer to a Immobilon-P Transfer Membrane, successive incubations with anti-CXCR-4 (1:500; Rabbit; Proteintech, USA), CXCR-7 (1:500; Rabbit; Proteintech, USA), p65 (0.5 µg/mL; Abcam, USA), p-p65 (0.5 µg/mL; Abcam, USA), and p-Iĸb (0.6 µg/mL; Santa Cruz Biotechnology, Inc., Santa Cruz, CA, USA) antibodies or anti-GAPDH antibody (Sangon Biotech) were performed, followed by corresponding horseradish peroxidase-conjugated secondary antibody (Sangon Biotech) incubation. The immunoreactive proteins were then detected using the ECL system (NCM Biotech, Suzhou, China). Bands were scanned using a densitometer (GS-700; Bio-Rad) and quantification was performed via Quantity One 4.6.3 software (Bio-Rad). Experiments were repeated at a minimum of three times.

### Cell proliferation assay

Cell proliferation assays were performed using Cell Counting Kit-8 (CCK-8; Dojindo, USA). Cells were plated in 96-well plates at 3 × 10^4^ cells per well and incubated for five days. Each day, 10 uL CCK-8 solution was added to each well. The OD value was read at 450 nm, 2 h after CCK-8 addition, according to the manufacturer instructions, and the cell viability was measured at intervals of 24 h. Experiments were repeated at a minimum of three times.

### Colony formation assay

Oral cancer cells (CAL 27, HN 30) were seeded at 1,000 cells per well in a 6-well plate and incubated overnight at 37 °C. The cells were treated with different concentrations of pDC-CM containing 10% FBS. Control wells were treated with DMEM containing 10% FBS alone. The plates were incubated for 14 days; during this period, the medium was changed twice per week with the appropriate concentration of pDC-CM. The plates were washed with ice-cold PBS, colonies were fixed with methanol for 15 min, stained with 2% crystal violet, and counted. Colonies consisting of ≥ 50 counts were scored. Experiments were repeated at a minimum of three times.

### Scratch wound healing assay

Cells were treated with different concentration of pDC-CM after creating a wound across the cell monolayer with a plastic tip. Cell migration into the wound surface was then measured every 6 h. Experiments were repeated at a minimum of three times.

### Cell migration and invasion assay

The cell migration assay was performed with transwell chambers (0.8 μm pores; Merck Millipore, USA), while the cell invasion assay was implemented by coating the upper surface of the transwell chambers with Matrigel (BD Biosciences, USA). Cells (3×10^4^) were resuspended in serum-free DMEM and different concentrations of pDC-CM were added to the interior of the inserts. DMEM containing 20% FBS was added to the lower chamber as a chemoattractant. Cells were incubated for 48 h at 37 °C in a CO_2_ incubator (5% CO_2_). Cells that migrated or invaded through the membrane were fixed and stained with crystal violet. Images of five randomly selected fields containing fixed cells were captured, and cells were counted. Experiments were repeated at a minimum of three times.

### Statistical analysis

Data are presented as mean ± standard deviation (SD) of at least three independent experiments. Comparisons of independent samples were performed using Student's *t*-test or nonparametric tests when appropriate (SPSS 11.0, Chicago). Correlation analyses were done using the Chi-square test or Spearman's rank correlation coefficient. Statistical significance was defined as *P* < 0.05 for all analyses.

## Results

### Increased pDC infiltration correlates with tumor growth and lymph node metastasis in OSCC patients

**Figure 1:**
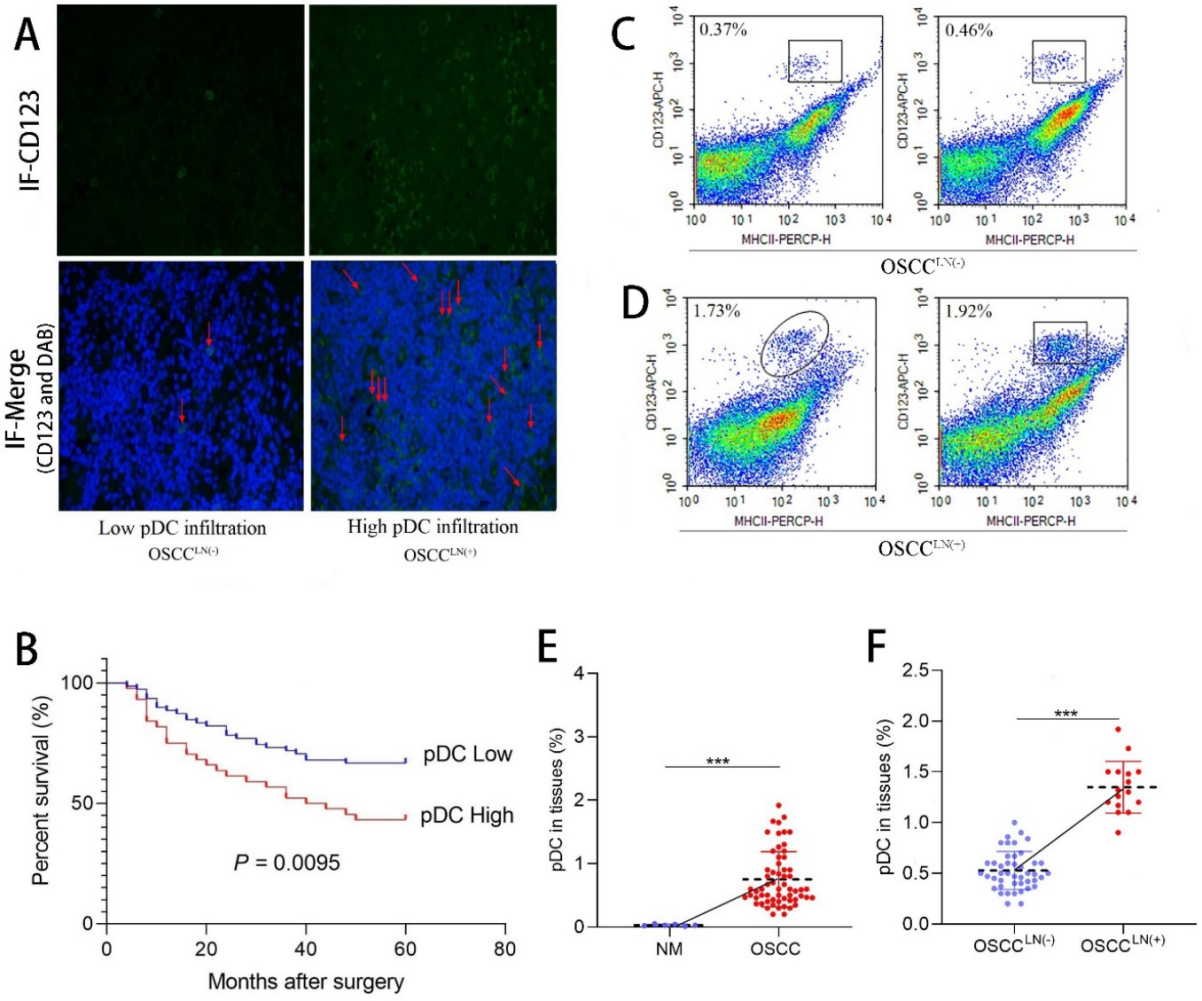
Tumor-infiltrating pDC in OSCC tissue. **(A)** Representative image of tumor-infiltrating pDC in OSCC tissue as visualized by immunofluorescence. (Low: < 10 pDC per HPF; high: > 10 pDC per HPF), Magnification: 400×. The red arrow indicates the stained cell of interest.** (B)** Cumulative survival rate of OSCC patients with low level and high level pDC infiltration.** (C)** Representative image of low infiltrated pDC in tumor tissue of OSCC^LN(-)^ patients by flow cytometry.** (D)** Representative image of high tumor-infiltrating pDC in tumor tissue of OSCC^LN(+)^ patients by flow cytometry. **(E)** Flow cytometry results showed tissue-infiltrating pDC were significantly higher in the OSCC tissue compared with normal oral mucosa.** (F)** Flow cytometry results showed tissue-infiltrating pDC were significantly higher in OSCC^LN(+)^ tissue compared with OSCC^LN(-)^ tissue. NM, Normal oral mucosa epithelium. OSCC^LN(-)^, OSCC patients without lymph node metastasis. OSCC^LN(+)^, OSCC patients with lymph node metastasis. *P < 0.05, ***P < 0.001.

We first evaluated the level of pDC infiltration in the paraffin-embedded tumor tissue sections of 122 OSCC patients using immunofluorescence. Results showed tumor-infiltrating pDC was positively correlated with tumor size (*P* = 0.045), and lymph node metastasis (*P* = 0.022) (**Table 1**). Significant increased tumor-infiltrating pDC was seen in carcinoma tissues of OSCC patients with lymph node metastasis (OSCC^LN(+)^) compared to that of without lymph node metastasis (OSCC^LN(-)^ ) (**Figure 1A**). More important, survival analysis of 122 OSCC patients demonstrated that increased tumor-infiltrating pDC was a strong predictor of poor outcome (*P* = 0.0095; **Figure 1B**). Further flow cytometry analysis from 60 freshly resected OSCC tissues and 6 normal oral mucosa also demonstrated a significantly higher pDC population (1.35 ± 0.25%) in carcinoma tissues of OSCC^LN(+)^ patients compared to OSCC^LN(-)^ patients. (0.53 ± 0.19%) (*P* < 0.01; **Figure 1C-F**).

### pDC-CM promote oral cancer cell proliferation and invasion *in vitro*

**Figure 2:**
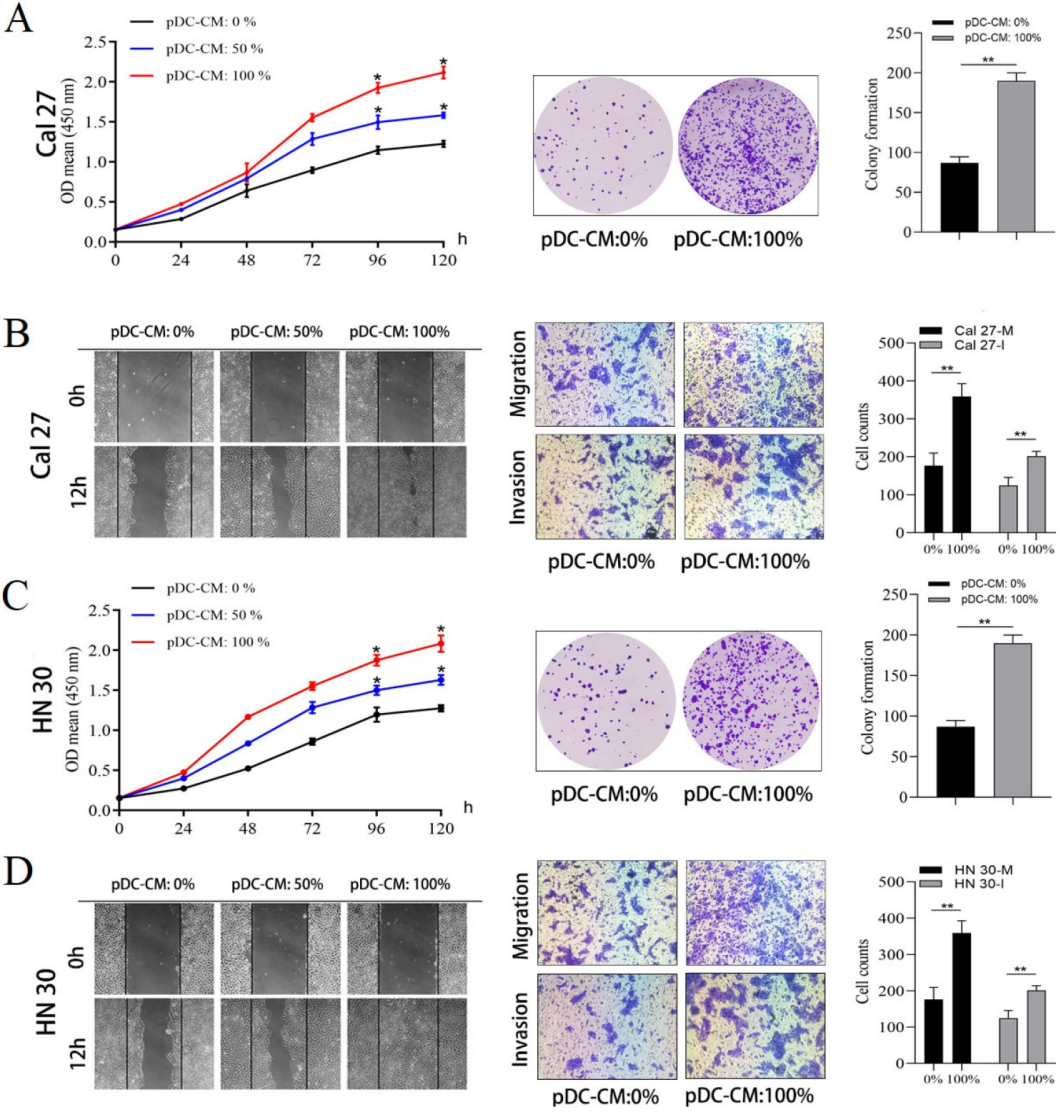
Tumor-infiltrating pDC promote oral cancer cell proliferation, migration and invasion *in vitro*. pDC were isolated and seeded at a final concentration of 5 × 10^5^ cells/mL and cultured overnight in serum-free DMEM at 37 °C and 5% CO_2_ to prepare pDC‑conditioned medium (pDC-CM). Oral cancer cells were stimulated with different concentrations of pDC-CM.** (A)** pDC-CM promote Cal 27 cell proliferation in a concentration-dependent manner as reflected by CCK-8 and colony formation assays.** (B)** pDC-CM promote Cal 27 cell migration and invasion as reflected by the scratch and transwell assays.** (C)** pDC-CM promote HN 30 cell proliferation in a concentration-dependent manner as reflected by CCK-8 and colony formation assays.** (D)** pDC-CM promote HN 30 cell migration and invasion as reflected by the scratch and transwell assays. The images from scratch assays were photographed every 6 h. Transwell assay magnification, 200 ×. Experiments were repeated at a minimum of three times. pDC-CM: 0%, DMEM containing 10% FBS; pDC-CM: 50%, Add an equal volume of DMEM containing 10% FBS to the original pDC-CM; pDC-CM: 100%, undiluted pDC-conditioned media. Cal 27(HN 30)-M/I, cells that migrated (M) or invaded (I) through the transwell chamber. *, *P* < 0.05. **, *P* < 0.01.

Next, the impact of tumor-infiltrating pDC on oral cancer cell was investigated. pDC were isolated and cultured overnight to prepare pDC‑conditioned medium (pDC-CM). The CCK-8 and colony formation assay revealed that pDC-CM significantly enhanced the rate of cell proliferation and colony formation in Cal 27 and HN 30 cell lines (*P* < 0.05;** Figure 2A**,** 2C**). Moreover, wound healing and transwell assays revealed that the migration and invasion capability of oral cancer cells treated with different concentrations of pDC-CM increased remarkably in comparison with that of untreated oral cancer cells (*P* < 0.01; **Figure 2B**,** 2D**), suggesting that tumor-infiltrating pDC have the ability to promote cancer cell proliferation, migration and invasion *in vitro*.

### Increased pDC infiltration positively correlates with CXCR-4 expression in OSCC

**Figure 3:**
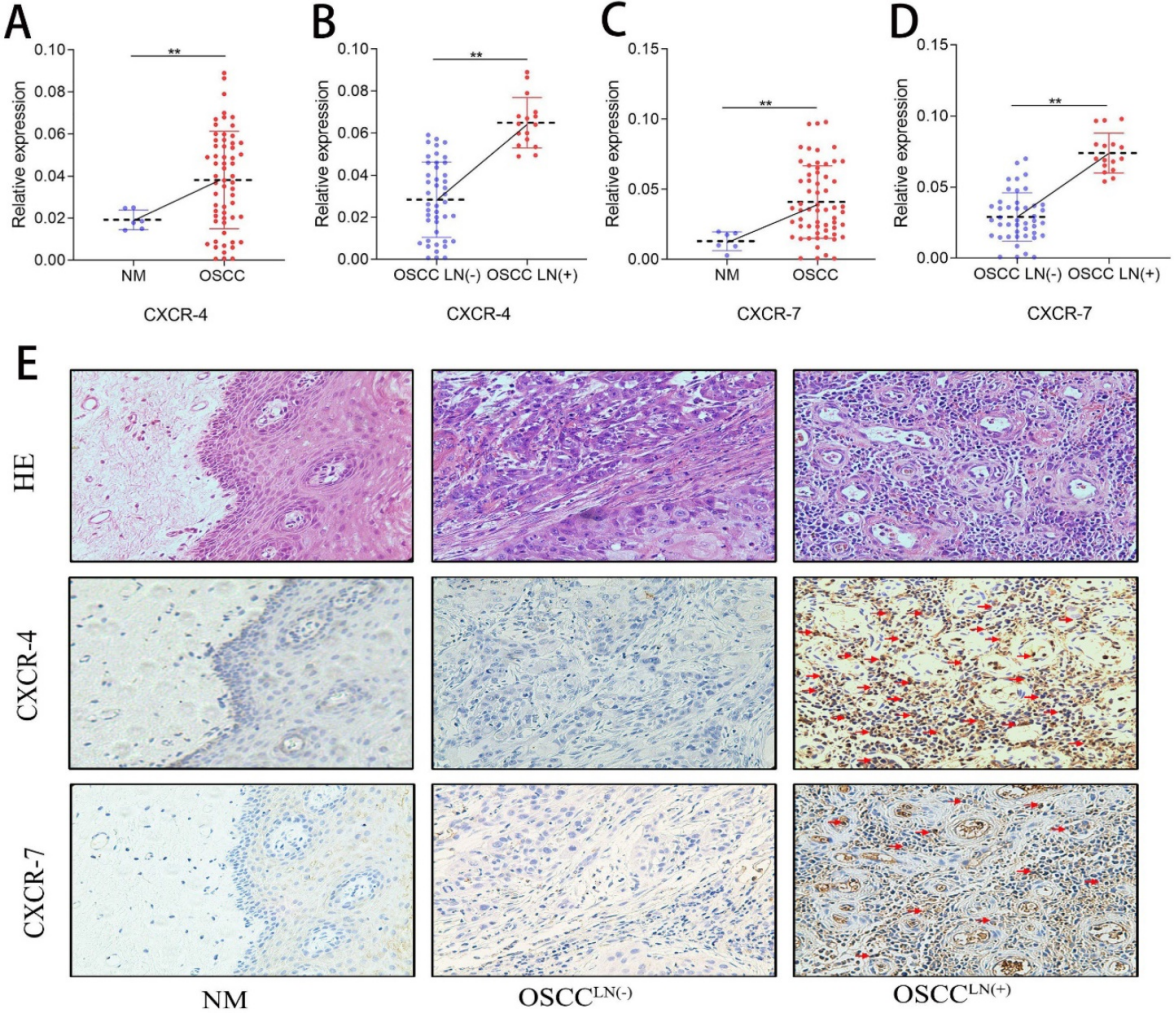
CXCR-4/7 expression in OSCC patients. **(A)** CXCR-4 mRNA expression was significantly higher in OSCC tissue compared with normal oral mucosa. **(B)** CXCR-4 mRNA expression was significantly greater in OSCC^LN(+)^ tissues compared with OSCC^LN(-)^ tissues. **(C)** CXCR-7 mRNA expression was significantly higher in the OSCC tissue compared with normal oral mucosa. **(D)** CXCR-7 mRNA expression was significantly greater in OSCC^LN(+)^ tumor tissues compared with OSCC^LN(-)^ tissues. **(E)** Representative image of HE and IHC staining for CXCR-4 and CXCR-7 in normal oral mucosa, OSCC^LN(-)^ and OSCC^LN(+)^ tumor tissue. Experiments were repeated at a minimum of three times. The red arrow indicates the stained cell of interest. NM, Normal oral mucosa epithelium; OSCC^LN(-)^, OSCC patients without lymph node metastasis; OSCC^LN(+)^, OSCC patients with lymph node metastasis. **, *P* < 0.01.

CXCR-4 and CXCR-7 mRNA expression of 60 freshly resected OSCC tissues and 6 normal oral mucosa were detected by qPCR. Results show that both CXCR-4 and CXCR-7 mRNA expression in the carcinoma tissue of OSCC^LN(+)^ patients were significantly higher than that in OSCC^LN(-)^ patients (**Figure 3A-D**). IHC detection also showed an increased CXCR-4 and CXCR-7 protein expression in carcinoma tissues of OSCC^LN(+)^ patients compared to that of OSCC^LN(-)^ patients (**Figure 3E**). In addition, we performed Spearman's rank correlation coefficient analysis and found a significant positive relationship between tumor-infiltrating pDC and CXCR-4 expression (r = 0.669, *P* < 0.01, **Supplemental Table 1**). However, such significance was not observed between tumor-infiltrating pDC and CXCR-7 (r = 0.024, *P* = 0.84,**Supplemental Table 1**), suggesting that CXCR-4 may play a more important role in pDC-mediated lymph node metastasis.

### CXCR-4 is involved in pDC-mediated oral cancer cell proliferation and invasion

**Figure 4:**
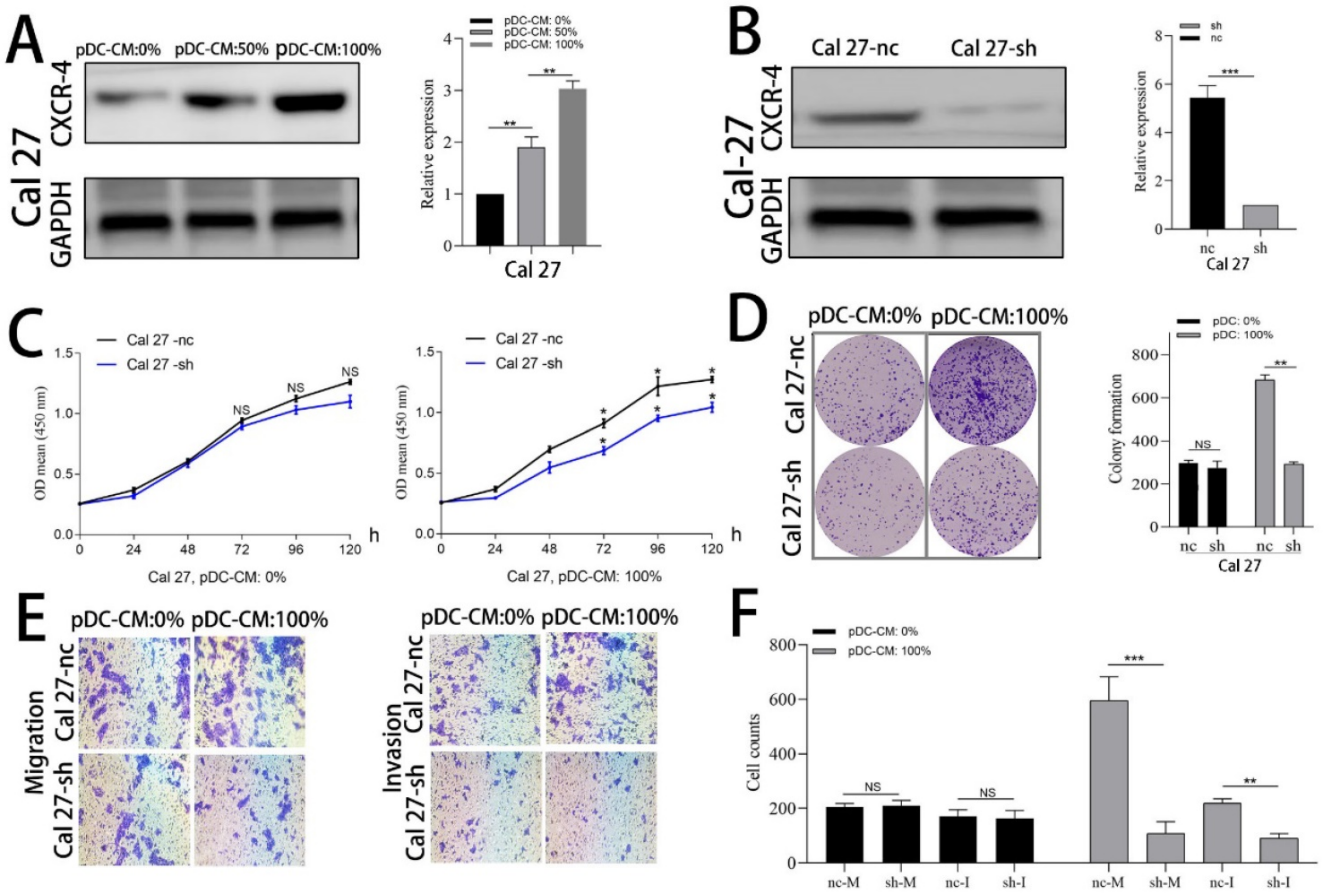
CXCR-4 is involved in pDC-mediated Cal 27 cells proliferation, migration and invasion. **(A)** Increased CXCR-4 expression was detected by western blotting after pDC-CM treatment in Cal 27 cells for 72 hours.** (B)** Cal 27 cells were cultured in 6-well cell culture plates and transfected with the lentiviraus-CXCR-4-shRNA(sh) or negative control (nc). CXCR-4 expression was further detected by western blotting after transfection. The effect of pDC-CM on Cal 27-nc and Cal 27-sh growth rates were assessed by CCK-8 **(C)** and colony formation assays **(D)**.** (E)** Representative image of cell migration and invasion by transwell assay.** (F)** Cal 27 cell migration (nc/sh-M) and invasion (nc/sh-I) were both decreased in the CXCR-4-silenced group upon pDC-CM treatment. NS, no significance. *, *P* < 0.05; **, *P* < 0.01; ***, *P* < 0.001.

Cal 27 and HN 30 cell lines were treated with pDC-CM for 72 h. Western blotting analysis showed that CXCR-4 expression of both Cal 27 and HN 30 were significantly increased in a concentration-dependent manner following pDC-CM treatment (**Figure 4A; Supplemental Fig. 1A**). We then used lentiviraus-CXCR-4-shRNA to silence CXCR-4 expression in Cal 27 (**Figure 4B**) and HN 30 (**Supplemental Fig. 1B**) cell lines and found that pDC-CM-mediated tumor cell proliferation was significantly inhibited in CXCR-4-silenced cells, as reflected by the CCK-8 (**Figure 4C; Supplemental Fig. 1C**) and colony formation (**Figure 4D; Supplemental Fig. 1D**) assays. In addition, Cells that migrated or invaded through the transwell chamber were also decreased in the CXCR-4-silenced cells upon pDC-CM treatment (*P* < 0.01; **Figure 4E, F; Supplemental Fig. 1E, F**), suggesting that CXCR-4 may play an important role in pDC-mediated cancer cell proliferation, migration and invasion.

### NF-κB activation contributes to pDC-mediated CXCR-4 overexpression in oral cancer cells

**Figure 5:**
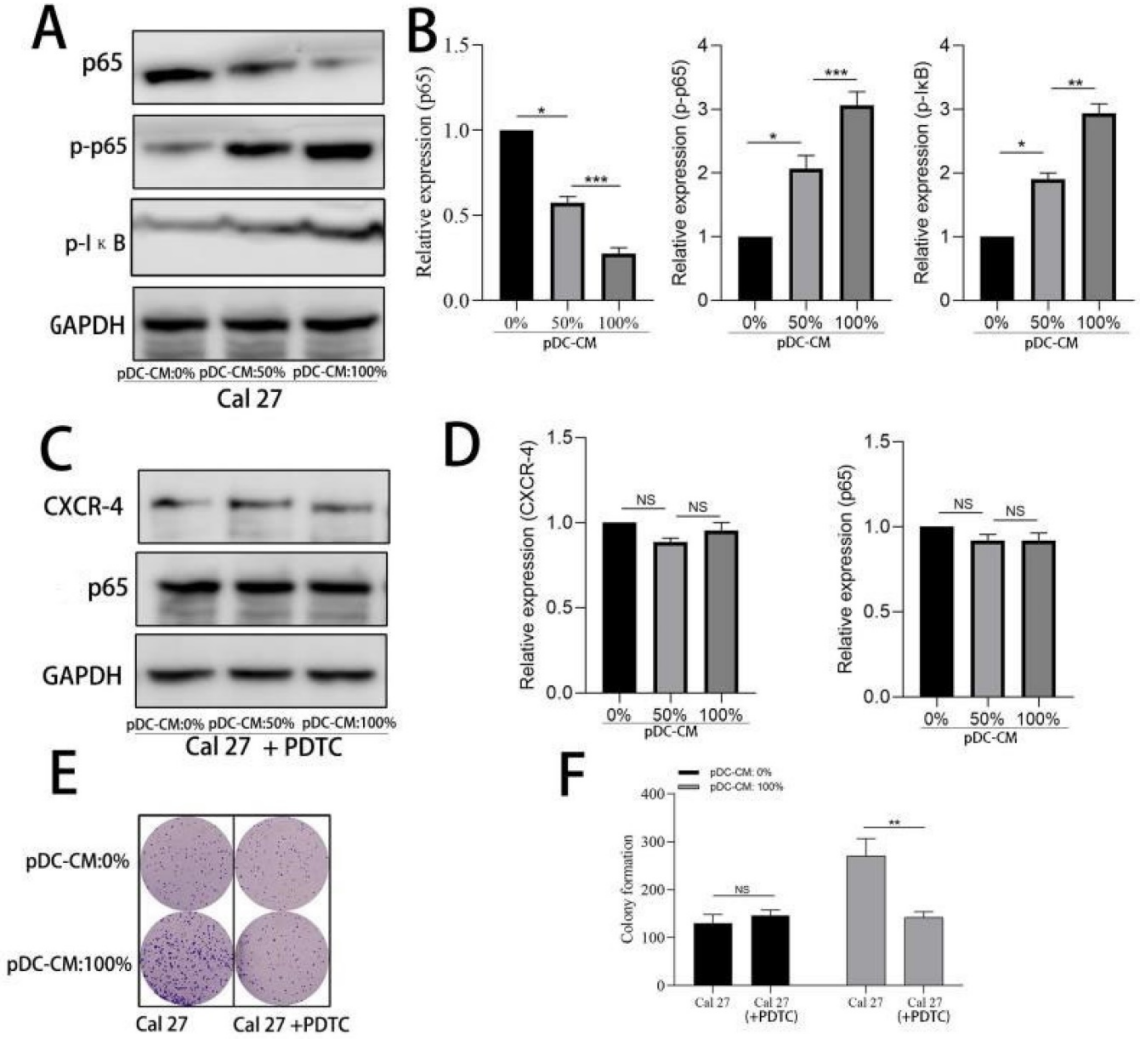
Activation of NF-κB in pDC-mediated CXCR-4 overexpression in oral cancer cells. **A/B.** Increased NF-κB activity in pDC-CM-treated Cal 27 cells assessed by western blotting.** C/D.** NF-κB inhibitor PDTC down-regulated pDC-CM-mediated CXCR-4 overexpression in Cal 27 cells.** E/F.** PDTC decreased pDC-CM-mediated oral cancer cell proliferation as reflected by colony formation assay. Experiments were repeated at least three times. *, *P* < 0.05. **, *P*< 0.01. ***, *P*< 0.001.

**Figure 6:**
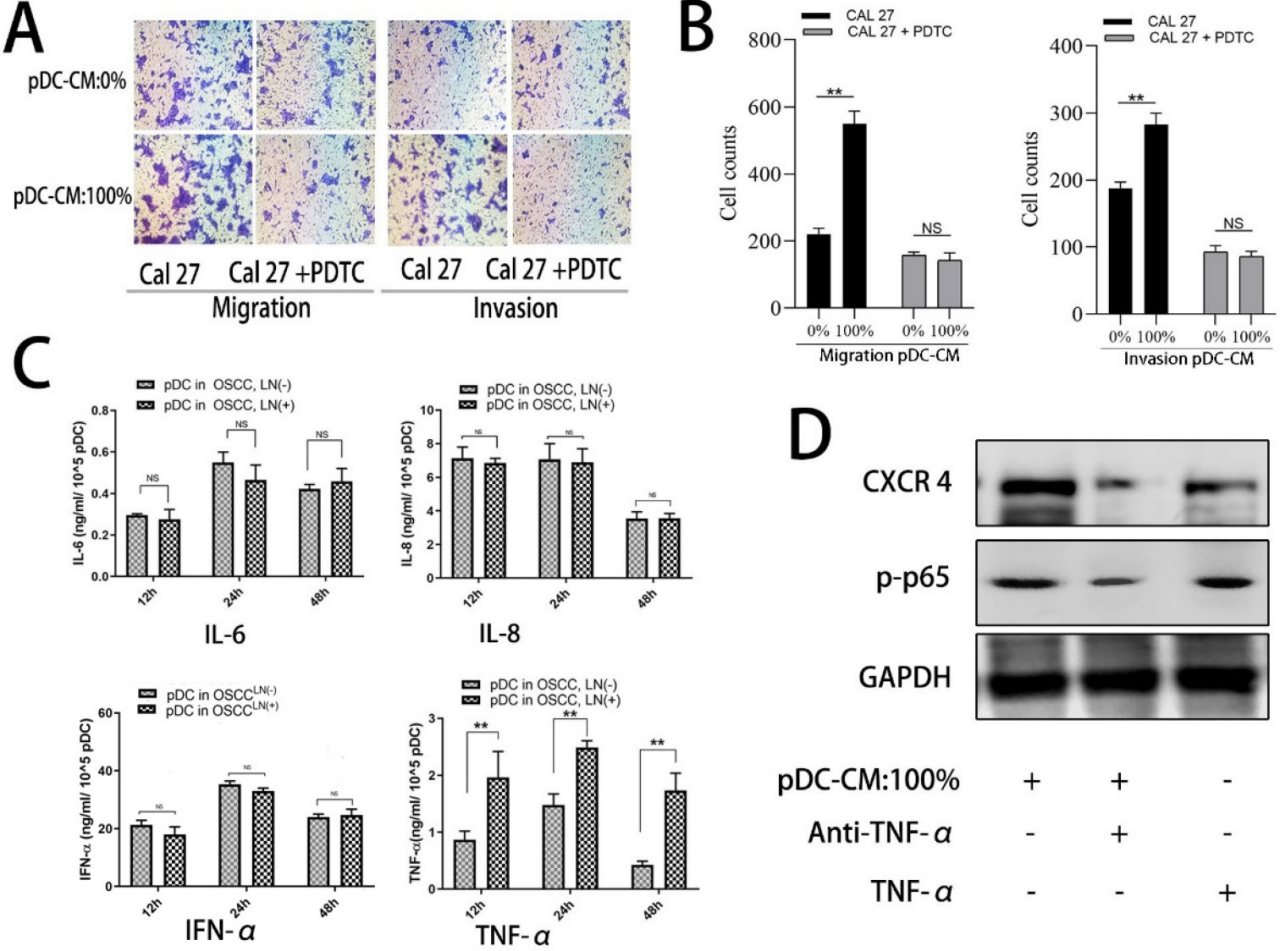
TNF-α is involved in pDC-mediated NF-κB activity in OSCC Cal 27 cells. **A/B.** NF-κB inhibitor, PDTC, decreased pDC-CM-mediated oral cancer Cal 27 cell migration and invasion as reflected by transwell assay.** C.** TNF-α secretion was higher in pDC-CM from OSCC^LN(+)^ patients than that in pDC-CM from OSCC^LN(-)^ patients.** D.** TNF-α neutralizing antibody partly reversed pDC-CM-mediated NF-κB activity and CXCR-4 overexpression in Cal27 cells as shown by western blotting analysis. The data are representative of at a minimum of three experiments. NS, No significance. **P < 0.01.

We further examined the activity of NF-κB pathway via western blotting. Results showed that NF-κB p65 was significantly decreased in the cytoplasm of Cal 27 cells treated with pDC-CM, while phosphorylated-p65 and phosphorylated IκB significantly increased following pDC-CM treatment (**Figure 5A, B**). Moreover, NF-κB inhibitor PDTC was then used in Cal 27 cells treated with pDC-CM, and resulted in no significantly change of CXCR-4 expression, NF-kB activation (**Figure 5C,D**), cancer cell proliferation (**Figure 5E,F**) and invasion (**Figure 6A,B**), suggesting that pDC-CM-mediated CXCR-4 overexpression in oral cancer Cal 27 cells occurs, in part, through the NF-κB pathway.

### pDC-mediated NF-κB activated CXCR-4 overexpression is partly induced by pDC-derived TNF-α secretion

To further decipher the mechanism by which pDC-CM induces NF-κB pathway activation, we chose four stimulating factors, including TNF-α, IL-6, IL-8, and IFN-α. ELISA results showed that the concentration of TNF-α in LN^+^ pDC-CM was higher than that in LN^-^ pDC-CM, suggesting that TNF-α secreted by tumor-infiltrating pDC may contribute to NF-κB activation in oral cancer cells (**Figure 6C**). We then used an anti-TNF-α antibody to neutralize pDC-CM-derived TNF-α, which caused significant inhibition of the pDC-CM-mediated NF-κB activity and CXCR-4 expression in Cal 27 cells (**Figure 6D**). Conversely, purified TNF-α treatment resulted in enhanced NF-κB activity and CXCR-4 expression (**Figure 6D**), which further suggests that TNF-α is an important mediator of tumor-infiltrating pDC in the promotion of tumor growth and metastasis.

## Discussion

Crosstalk between tumor cells and tumor-infiltrating immune cells is extremely important for cancer development and treatment outcome 25. Cancer cells can secrete immunomodulatory cytokines that act on immune cells, impairing their function and facilitating tumorigenesis 26. Accordingly, these infiltrated immune cells can also promote tumor progression and metastasis through communication with cancer cells via secreted growth factors that directly affect cell motility, and chemokines that induce cancer cell migration to specific organs 27. Previously, our study provided strong evidence that oral cancer cells can secrete immunosuppressive mediators, such as TGF-β and IL-10, which in turn lead to the impaired function of tumor-infiltrating pDC 20. The present study shows that these impaired pDC in the pre-tumor microenvironment can also promote cancer cell proliferation and migration via NF-κB-activated CXCR-4 overexpression by a paracrine mechanism.

Metastasis to regional LNs is an important step in cancer progression and is commonly used to predict poor clinical outcome 28. Accumulating evidence suggests that tumor-infiltrating immune cells are closely related to LN metastasis. For example, increased CD8^+^ T cell and M1 TAM infiltration are often correlated with a low incidence of LN metastasis, while increased regulatory T cells and M2 TAMs primarily indicate a high incidence of LN metastasis 5. However, the relationship between tumor-infiltrating pDC and LN metastasis is rarely reported. In 2017, we first reported that tumor-infiltrating pDC are closely related to LN metastasis in OSCC patients 19. Similarly, this year, Gadalla et al. also found a significant increase in tumor-infiltrating pDC in breast cancer tissues of LN^+^ patients compared to that of LN^-^ patients 29. Unfortunately, none of these studies clarified whether tumor-infiltrating pDC are effective as a predictor for LN metastasis based on a large clinic sample. In the present study, we collected clinicopathological data and follow-up information from 122 OSCC patients to confirm the predicted role of tumor-infiltrating pDC in oral cancer LN metastasis.

In the present study, we interestingly found that tumor-infiltrating pDC were highly present in OSCC tissues expressing high levels of CXCR-4. We speculated that there may be an interaction between pDC infiltration and CXCR-4 expression in the oral cancer microenvironment. CXCR-4, one of the chemokine receptors belonging to the CXC receptor family, plays a significant role in cancer cell proliferation and migration 30. High levels of CXCR-4 were found in breast cancer cells, which is believed to indicate the metastatic destination of tumor cells 31. Loss of cell surface CXCR-4 expression on hematopoietic stem cells is associated with decreased migration activity 32. In oral cancer, CXCR-4 has been found to be upregulated in LN metastatic OSCC cell lines compared with nonmetastatic cell lines 33. Moreover, CXCR-4 stably transfected in oral cancer cells frequently induces metastasis to cervical LN in nude mice 34. In parallel with these results, we confirmed an increased expression of CXCR-4 in primary oral cancer patient tumors with LN metastases. Importantly, we also demonstrated that pDC-CM could significantly promote OSCC proliferation and migration via increasing CXCR4 expression *in vitro*, as reflected by the colony formation and transwell assays, suggesting that tumor-infiltrating pDC may contribute to the overexpression of CXCR-4, and subsequent growth and metastasis in oral cancer.

The signal transduction pathways that modulate the activity of CXCR-4 transcription factors are highly diverse. A number of which, including the nuclear respiratory factor-1, SP-1, NF-κB, and activator protein-1 pathways, have been reported to be responsible for constitutive and inducible expression of CXCR-4 35. The present study provides sufficient evidence to suggest direct involvement of NF-κB in the regulation of CXCR-4 expression in OSCC. Similarly, a study showed that constitutively active NF-κB upregulates CXCR-4 expression and promotes tumor cell migration and metastasis in breast cancer cells 36. Another study in cervical cancer also found that NF-κB promotes cancer cell migration to the lung via upregulation of CXCR-4 expression 37. Therefore, our results, as well as those published by others, support the therapeutic benefits of NF-κB inhibitors for metastatic cancer patients.

Several reports have described the secretion of various factors by tumor-infiltrating immune cells, including TNF-α, IL-10, and TGF-β, as impacting the NF-κB pathway 38. However, it has yet to be clearly established which factors have an important role in pDC-CM-mediated NF-κB activation in oral cancer. Interestingly, our ELISA results indicate a higher secretion of TNF-α in pDC isolated from LN^+^ cancer tissue in comparison with that of pDC from LN^-^ cancer tissue, suggesting that the specific stimulating effect of the pDC-derived factor on NF-κB activation and subsequent CXCR-4 overexpression may be partly mediated through TNF-α secretion. TNF-α is reported to directly activate NF-κB pathways through the stimulation of TNF receptors 39. Simultaneously, blocking TNF-α in the tumor microenvironment significantly down-regulates NF-κB activity and reduces cancer cell proliferation and invasion in breast cancer 40. To further support our hypothesis, we showed that direct TNF-α stimulation upregulates CXCR-4 expression in an oral cancer Cal 27 cell line via NF-κB activation, and addition of TNF-α neutralizing antibodies could decrease the pDC-CM-mediated NF-κB activation. Therefore, we speculated that pDC-derived TNF-α contributed in part, along with other inflammatory mediators in the tumor microenvironment, to activate the NF-κB pathway.

In conclusion, our data provide strong evidence that impaired tumor-infiltrating pDC are a strong predictor for LN metastasis in oral cancer, and that increased tumor-infiltrating pDC contributes to upregulation of CXCR-4 expression, possibly through TNF-α-mediated NF-κB activation, which ultimately leads to cell proliferation and migration toward LN via the CXCR4/SDF-1 chemoattraction axis. These data support the notion that crosstalk between tumor cells and tumor-infiltrating immune cells is critical for cancer development and metastasis. Careful manipulation of these impaired tumor-infiltrating pDC may help develop an important alternative immunotherapy for oral cancer.

## Supplementary Material

Supplementary figures and tables.

## Figures and Tables

**Table 1:** Correlations between tumor-infiltrating pDC and clinicopathologic parameters of OSCC patients (N=122)

Clinicopathologic parameters	Tumor-infiltrating pDC		*P*-value
	Low (%) (N=78)	High (%) (N=44)	
**Age (year)**			0.563
<50	33 (27.0%)	21 (17.2%)	
≥50	45 (36.9%)	23 (18.9%)	
**Gender**			0.187
Male	40 (32.8%)	28 (23.0%)	
Female	38 (31.1%)	16 (13.1%)	
**Alcohol use**			0.729
Yes	38 (31.1%)	20 (16.4%)	
No	40 (32.8%)	24 (19.7%)	
**Smoking**			0.289
Yes	33 (27.0%)	23 (18.9%)	
No	45 (36.9%)	21 (17.2%)	
**Tumor size**			0.045*
T1/T2	50 (41.0%)	20 (16.4%)	
T3/T4	28 (23.0%)	24 (19.7%)	
**Lymph node metastasis**			0.022*
Yes	31 (25.4%)	27 (22.1%)	
No	47 (38.5%)	17 (13.9%)	
**Distant metastasis**			0.965
Yes	18 (14.8%)	10 (8.2%)	
No	60 (49.2%)	34 (27.9%)	
**Tumor differentiation**			0.187
Well	40 (32.8%)	28 (23.0%)	
Moderate/poorly	38 (31.1%)	16 (13.1%)	

*, *P* < 0.05.
