# Supplementary material for: Increased tumor-infiltrating plasmacytoid dendritic cells promote cancer cell proliferation and invasion via TNF-α/NF-κB/CXCR-4 pathway in oral squamous cell carcinoma
Source: J Cancer. 2021 Mar 19;12(10):3045–56. doi: 10.7150/jca.55580 (PMC8040884; doi:10.7150/jca.55580)
Supplement: Supplementary file 1 — Supplementary figures and tables. [file jcav12p3045s1.pdf]

Supplemental Table 1 Spearman's rank correlation coefficient analysis between tumor-infiltrating pDC and CXCR-4/7 expression.

Correlation coefficient

			pDC	CXCR-4
Spearman Rho	pDC	R	1.000	.669**
		Sig. (two tailed)	.	.000
		N	66	66
	CXCR-4	R	.669**	1.000
		Sig. (two tailed)	.000	.
		N	66	66

**. correlation significant at 0.01 level

Correlation coefficient

			pDC	CXCR-7
Spearman Rho	pDC	R	1.000	.024
		Sig. (two tailed)	.	.84
		N	66	66
	CXCR-7	R	.024	1.000
		Sig. (two tailed)	.84	.
		N	66	66

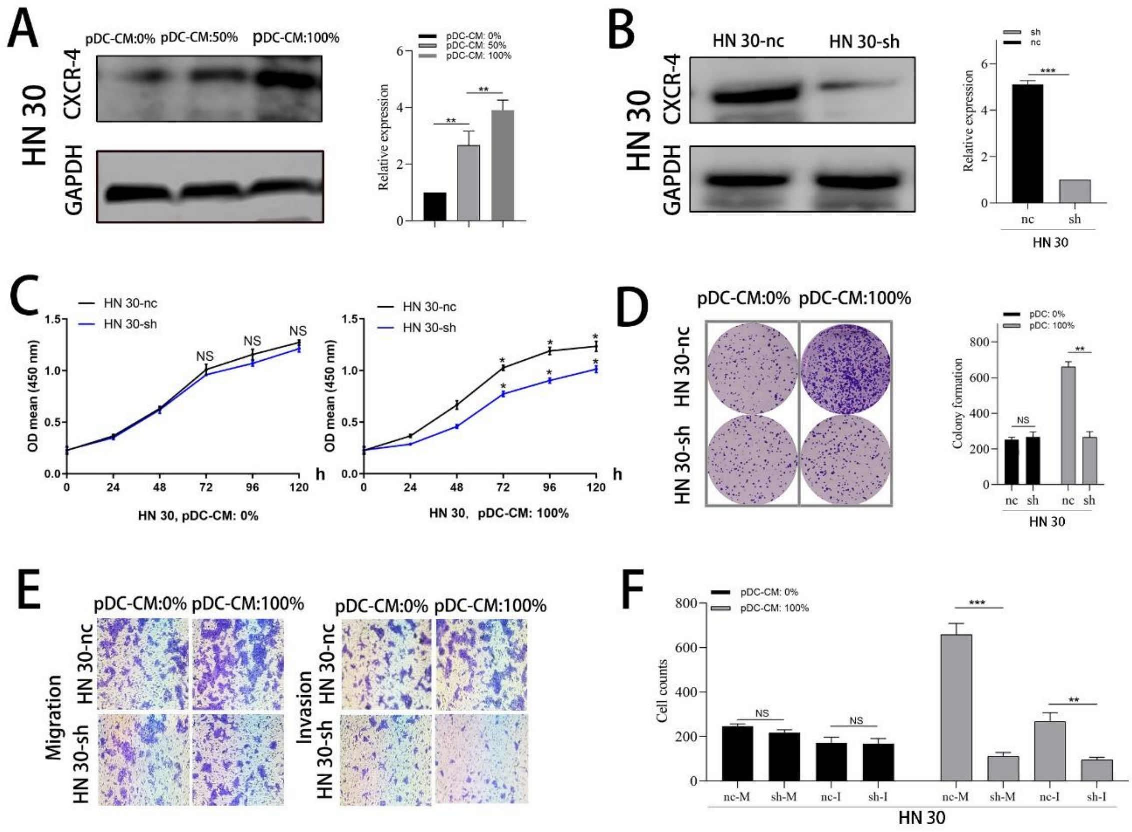

Supplemental Figure 1. CXCR-4 is involved in pDC-mediated HN 30 cells proliferation, migration and invasion. **(A)** Increased CXCR-4 expression was detected by western blotting after pDC-CM treatment in HN 30 cells for 72 hours. **(B)** HN 30 cells were cultured in 6-well cell culture plates and transfected with the lentiviraus-CXCR-4-shRNA(sh) or negative control (nc). CXCR-4 expression was further detected by western blotting after transfection. The effect of pDC-CM on HN 30-nc and HN 30-sh growth rates were assessed by CCK-8 **(C)** and colony formation assays **(D)**. **(E)** Representative image of cell migration and invasion by transwell assay. **(F)** HN 30 cell migration (nc/sh-M) and invasion (nc/sh-I) were both decreased in the CXCR-4-silenced group upon pDC-CM treatment. NS, no significance. *, $P < 0.05$; **, $P < 0.01$; ***, $P < 0.001$.

See below for STR results of cell lines

1、 Sample ID：CAL27

2、 Original Material：Cell pellets

3、 Check time:2019-7-4

4、 Methods：

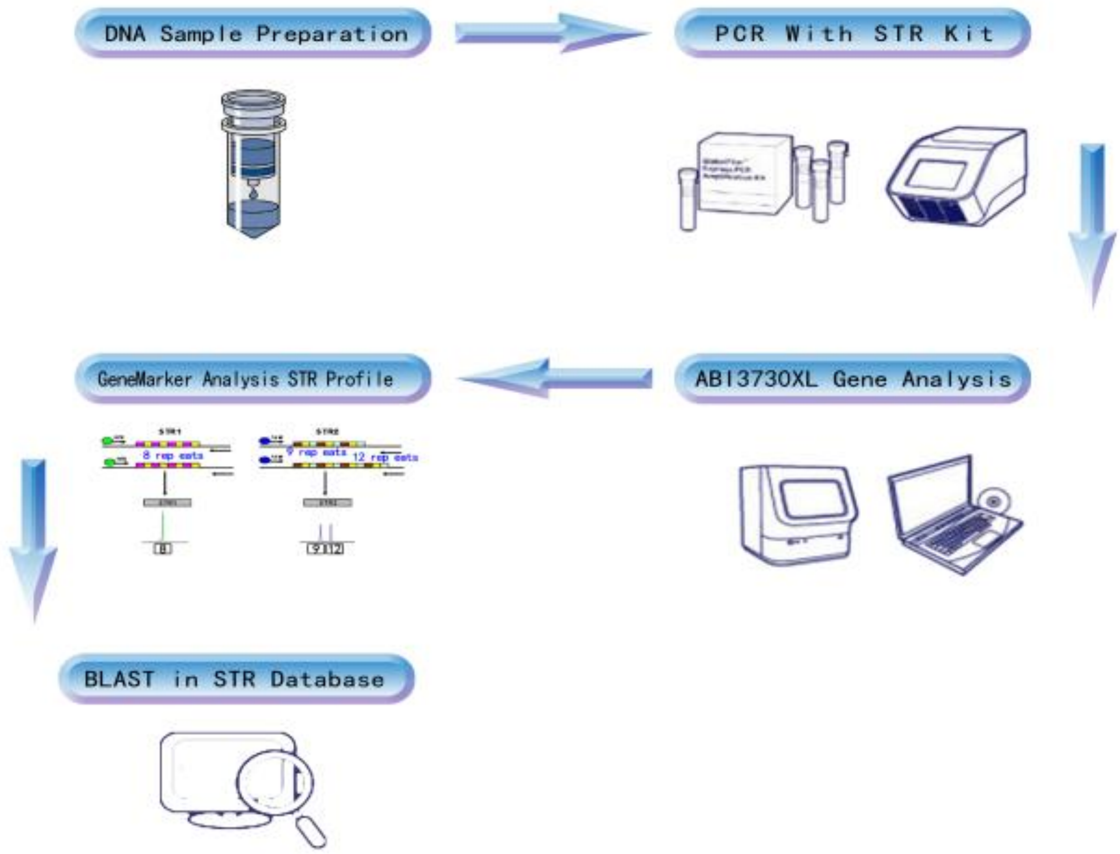

5、 Results：

Negative and positive test results are correct.

Amplification map of Genomic DNA clear，Genotyping results well.

STR Profile :

Genetic Site	Customer sample		**ATCC**	
	CAL27		CAL27	
Amelogenin	X	X	X	X
CSF1PO	10	12	10	12
D13S317	10	11	10	11
D16S539	11	12	11	12
D5S818	11	12	11	12
D7S820	10	10	10	10
THO1	6	9.3	6	9.3
TPOX	8	8	8	8
vWA	14	17	14	17
Percent match between the sample and the database profile：**100%**				

6、 Summary：

细胞的 STR 位点和 Amelogenin 位点的基因分型结果						
Loci	送检细胞 STR 信息			细胞库细胞 STR 信息		
	送检细胞名：HN30			细胞库细胞名：WSU-HN30		
	Allele1	Allele2	Allele3	Allele1	Allele2	Allele3
D5S818	12	13		12	13	
D13S317	11	11		11	11	
D7S820	10	10		10	10	
D16S539	11	12		11	12	
VWA	15	18		15	18	
TH01	7	9		7	9	
AMEL	X	Y		X	Y	
TPOX	6	8.3	11	6	11	
CSF1PO	10	12		10	12	
D12S391	17	19				
FGA	20	24				
D2S1338	21	22	25			
D21S11	28	28				
D18S51	13	16				
D8S1179	15	17				
D3S1358	16	16				
D6S1043	11	20				
PENTAE	8	12				
D19S433	13	17				
PENTAD	3.2	3.2				
D1S1656	14	17				

上海翼和应用生物技术有限公司联系地址：上海市松江区龙腾路 1015 弄中星创意园 2 号 502 室
网址：http://www.biowing.com.cn/邮箱：market@biowing.com.cn 服务热线：021-33559491
